# Supplementary material for: Comparative Efficacy and Safety of Glucagon-like Peptide-1 Receptor Agonists in Children and Adolescents with Obesity or Overweight: A Systematic Review and Network Meta-Analysis
Source: Pharmaceuticals (Basel). 2024 Jun 24;17(7):828. doi: 10.3390/ph17070828 (PMC11279917; doi:10.3390/ph17070828)
Supplement: Supplementary file 1 [file pharmaceuticals-17-00828-s001.zip › pharmaceuticals-3027667-supplementary.pdf]

Supplementary Table S1. Characteristics of included studies.

First author, year	Country	Design	Population	Intervention	Control	Dose regimen
Kelly, 2013	United States	Randomized, double-blind, placebo controlled, multicenter clinical trial	Severe obesity	13 weeks of exenatide	Volume-matched placebo pen	Initiated at 5 mcg twice daily. After 1 months, up titrated to 10 mcg twice daily for the remaining 2 months
Kelly, 2012	United States	Crossover RCT	Extreme obesity	13 weeks of exenatide	Volume-matched placebo	Initiated at a dose of 5 mcg twice daily. Up titrated to 10 mcg twice daily after 1 month for the remainder of the trial. If the 10-mcg dose was not tolerated, the dose was reduced to 5 mcg.
Danne, 2017	Germany	Randomized, double-blind, parallel-group, placebo-controlled trial	Obesity	5 weeks of liraglutide	Volume-matched placebo pen	Initiated at a dose of 0.6 mg, and was increased by 0.6 mg/week to a maximum of 3.0 mg/day
Klein, 2014	Belgium Slovenia United Kingdom US	Double-blind, randomized, placebo-controlled, parallel group trial	Overweight and type 2 diabetes	5 weeks of liraglutide	Volume-matched placebo pen	Initiated at 0.3 mg daily for first week and increased weekly thereafter to 0.6 mg, 0.9 mg, 1.2 mg, and 1.8 mg.
Mastrandrea, 2018	United States	Randomized, double-blind, placebo-controlled trial	Obesity	7 weeks to 13 weeks of liraglutide	Equal volume of placebo	Escalated from 0.3 to 1.2 mg in weekly increments of 0.3 mg and then followed with 0.6-mg weekly increments to a maximum dose of 3.0 mg or maximum tolerated dose
Arslanian, 2022	Nine countries	Phase 3, randomized, placebo-controlled, parallel-group, superiority trial	Overweight and type 2 diabetes	26 weeks dulaglutide	Visually identical, single-use, single-dose pen devices	Initiated at the 0.75-mg dose for the first 4 weeks and then escalated to the 1.5-mg dose if the participant did not have unacceptable side effects with the lower dose.
Weghuber, 2020	Sweden; Austria	Parallel, double-blinded, randomized, placebo controlled two-arm study	Obesity	26 weeks of Exenatide	Volume-matched placebo pen	Weekly subcutaneous injections of the exenatide 2 mg
Tamborlane, 2019	84 sites in 25 countries.	Randomized, parallel-group, placebo-controlled trial	Overweight and type 2 diabetes	52 weeks liraglutide	Visually identical prefilled pen injectors	liraglutide was initiated at a dose of 0.6 mg per day and was escalated in both groups in increments of approximately 0.6 mg each week over the course of 2 to 3 weeks.
Fox, 2022	United States	Randomized, double-blind, placebo-controlled trial	Severe obesity	52 weeks of exenatide	Matching placebo devices	Initiated and maintained at a dose of 2.0 mg weekly

Kelly, 2020	Belgium	Randomized, double-blind, placebo-controlled, phase 3 trial	Obesity	56 weeks of liraglutide	Volume-matched placebo pen	Initiated at a dose of 0.6 mg daily for 1 week, increased weekly thereafter until the maximum tolerated dose or 3.0 mg daily.
Weghuber, 2022	Austria, Belgium, Croatia, Ireland, Mexico, Russian Federation, United Kingdom, United States	Multinational, double-blind, parallel-group, randomized, placebo-controlled, phase 3a	Obesity	68 weeks of semaglutide	Matching placebo	Initiated at a dose of 0.25 mg once weekly for the first 4 weeks, followed by escalation every 4 weeks to 0.5, 1.0, 1.7, and 2.4 mg.

Supplemental Figure S1. Risk of bias assessment.

A. Breakdown of risk of bias assessment across individual studies.

Study, first author	Year	Bias arising from randomization process	Bias due to deviations from intended interventions	Bias due to missing outcome data	Bias in measurement of the outcome	Bias in selection of the reported result	Overall
Arslanian	2022	+	+	+	+	+	+
Danne	2017	?	+	+	+	?	?
Fox	2022	+	+	?	+	+	?
Kelly	2013	?	+	?	+	+	?
Weghuber	2022	+	+	-	+	+	-
Tamborlane	2019	+	+	?	+	+	?
Weghuber	2020	+	+	-	+	+	-
Kelly	2012	?	-	-	+	+	-
Klein	2014	?	+	+	+	+	?
Kelly	2020	+	+	-	+	+	-
Mastrandrea	2019	?	+	-	+	+	-

B. Summary of risk of bias scores across categories.

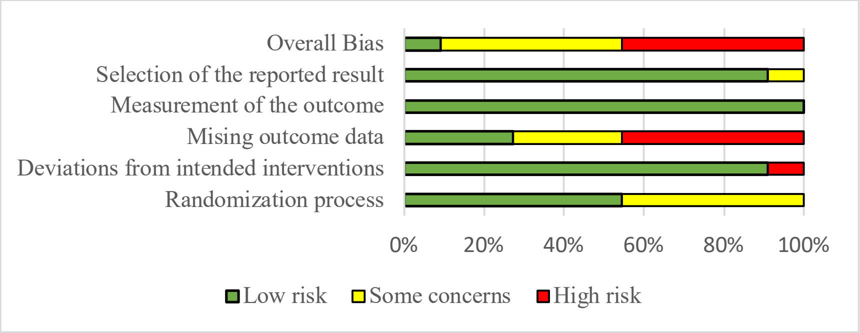

Supplemental Figure S2. Network graphs of each outcome from subgroup analysis. (a) Weight, (b) Waist, (c) BMI, and (d) BMI z score.

(a) Weight:

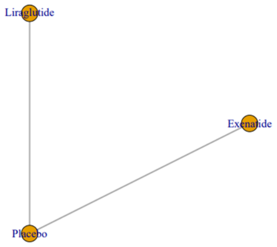

All, < 26 weeks (4 studies)

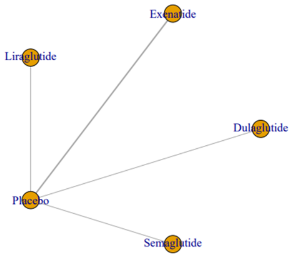

All, ≥26 weeks (5 studies)

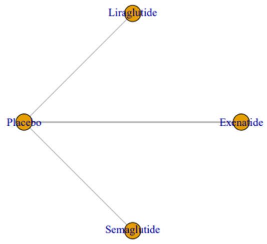

Obesity, ≥ 26 weeks (4 studies)

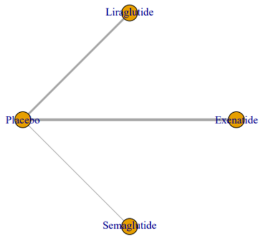

Obesity, all duration (8 studies)

(b) Waist

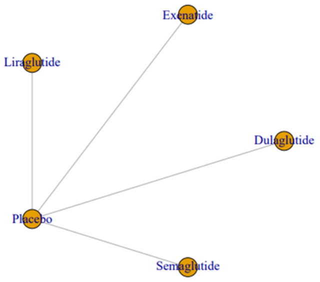

All population, ≥26 weeks (4 studies)

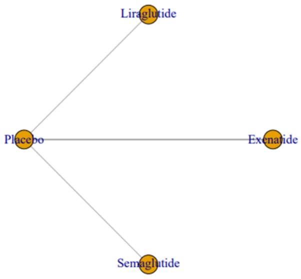

Obesity, all duration (4 studies)

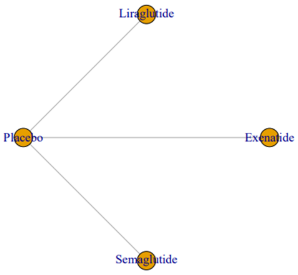

Obesity, ≥ 26 weeks (3 studies)

(c) BMI (4 studies)

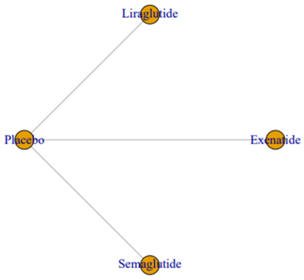

Obesity, ≥ 26 weeks (5 studies)

(d) BMI z score

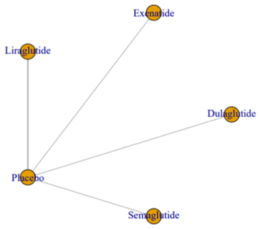

All, ≥ 26 weeks (5 studies)

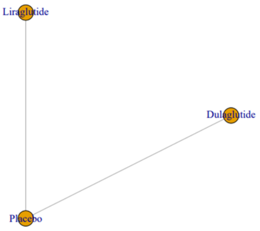

Diabetes (2 studies)

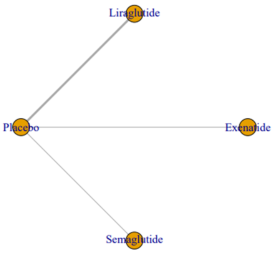

Obesity, all duration (5 studies)

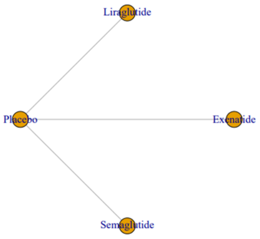

Obesity, ≥ 26 weeks (3 studies)

Supplemental Figure S3. League tables of other outcome analyses

A. A1c and FPG

Dulaglutide	−0.95 (−2.40, 0.59)	−1.35 (−2.93, 0.26)	−1.08 (−2.90, 0.85)	**−1.39 (−2.71, −0.04)**

0.11 (−3.28, 3.31)	Liraglutide	−0.39 (−1.50, 0.68)	−0.13 (−1.54, 1.26)	−0.44 (−1.13, 0.15)
2.12 (−2.03, 5.84)	1.95 (−1.30, 5.34)	Exenatide	0.26 (−1.27, 1.85)	−0.05 (−0.95, 0.81)
/	/	/	Semaglutide	−0.32 (−1.56, 0.93)
1.96 (−0.85, 4.76)	**1.87 (0.247, 3.67)**	−0.12 (−2.96, 2.77)	/	Placebo

B. SBP and DBP

Exenatide	−2.03 (−7.91, 3.34)	−1.38 (−8.34, 5.65)	−3.29 (−7.37, 0.61)
1.69 (−3.14, 6.13)	Liraglutide	0.65 (−5.99, 8.14)	−1.23 (−5.46, 2.97)
0.747 (−4.72, 6.0)	−0.93 (−6.18, 4.35)	Semaglutide	−1.91 (−7.82, 3.66)
1.34 (−2.12, 4.63)	−0.31 (−3.26, 2.88)	0.62 (−3.70, 4.95)	Placebo

C. Insulin and QOL

Liraglutide	−0.30 (−13.7, 13.5)	/	−3.17 (−15.6, 8.66)
−5.42 (−15.5, 4.69)	Exenatide	/	−2.98 (−9.45, 3.45)
3.03 (−5.61, 11.8)	8.38 (−1.91, 18.5)	Semaglutide	/
−1.32 (−7.32, 4.59)	4.12 (−3.75, 12.1)	−4.27 (−10.8, 2.18)	Placebo

A1c
FPG
SBP
DBP
Insulin
QOL

The league tables show the relative effects of each medication (the treatment on the column to the treatment of the row). The relative effects are measured as a difference in mean difference (DMD) with 95%CI for mean change in A1c, FPG, SBP, DBP, insulin, and quality-of-life. Bold indicates statistical significance. Abbreviation: A1c, hemoglobin A1C; DBP, Diastolic blood pressure; SBP, Systolic blood pressure; FPG, Fasting plasma glucose; QOL, Quality of life.

Supplemental Figure S4. League tables of subgroup analysis of main outcomes A.

Weight

(a) Overall population (Obesity and overweight)

Dulaglutide	/	/	/	/
−4.38 (−28.92, 19.17)	Liraglutide	2.19 (−2.27, 6.63)	/	−1.26 (−4.22, 1.98)
−3.54 (−24.16, 17.06)	0.91 (−19.66, 20.42)	Exenatide	/	**−3.48 (−6.61, −0.29)**
−17.71 (−43.25, 6.45)	−13.34 (−38.48, 10.60)	−14.13 (−37.48, 6.70)	Semaglutide	/
0.07 (−17.14, 17.04)	4.50 (−12.09, 20.39)	3.62 (−8.47, 15.16)	**17.72 (0.59, 35.74)**	Placebo

(b) Obesity alone:

Liraglutide	1.19 (−2.21, 4.48)	**15.4 (9.79, 21.0)**	−2.29 (−4.63, 0.05)
0.97 (−19.9, 21.9)	Exenatide	**14.3 (8.68, 19.6)**	**−3.47 (−5.82, −1.24)**
−13.3 (−37.4, 9.93)	−14.3 (−34.9, 7.19)	Semaglutide	**−17.7 (−22.8, −12.7)**
4.43 (−12.7, 21.7)	3.47 (−8.77, 16.2)	**17.7 (0.66, 35.0)**	Placebo

≥26 weeks
<26 weeks
All duration

B. BMI z score

Dulaglutide	/	/	/	/
−0.11 (−1.32, 1.20)	Liraglutide	−0.12 (−2.02, 1.57)	0.77 (−1.03, 2.60)	−0.23 (−1.51, 1.03)
−0.07 (−1.52, 1.42)	0.03 (−1.24, 1.29)	Exenatide	0.92 (−0.84, 2.76)	−0.09 (−1.32, 1.20)
−0.98 (−2.41, 0.50)	−0.88 (−2.17, 0.35)	−0.91 (−2.39, 0.59)	Semaglutide	−1.00 (−2.31, 0.28)
0.011 (−1.00, 1.04)	0.12 (−0.61, 0.82)	0.09 (−0.96, 1.13)	1.00 (−0.03, 2.10)	Placebo

Dulaglutide	/	/	/	/
−0.04 (−0.20, 0.12)	Liraglutide	−0.07 (−0.96, 0.84)	0.83 (−0.09, 1.66)	−0.17 (−0.62, 0.26)
/	/	Exenatide	0.91 (−0.14, 1.92)	−0.09 (−0.88, 0.61)
/	/	/	Semaglutide	**−1.00 (−1.74, −0.22)**
0.01 (−0.10, 0.12)	0.05 (−0.07, 0.17)	/	/	Placebo

≥26 weeks, all population
≥26 weeks, obesity
≥26 weeks, Diabetes
All duration, Obesity

C. BMI

Liraglutide			
−0.02 (−9.85, 8.27)	Exenatide		
−4.35 (−14.9, 6.29)	−4.42 (−13.0, 6.26)	Semaglutide	
1.63 (−5.61, 8.97)	1.61 (−3.14, 8.07)	5.99 (−1.63, 13.3)	Placebo

≥26 weeks, obesity

D. Waist circumference:

Liraglutide	−0.71 (−15.7, 14.1)	9.10 (−7.65, 26.0)	−3.00 (−15.3, 8.69)
−0.14 (−21.8, 21.1)	Exenatide	9.79 (−4.54, 25.6)	−2.27 (−10.9, 6.27)
−9.13 (−30.9, 11.5)	−8.88 (−29.4, 12.3)	Semaglutide	**−12.1 (−24.6, −0.32)**

2.95 (−12.4, 18.0)	3.12 (−12.3, 19.0)	12.1 (−3.42, 27.7)	Placebo

Liraglutide				
−0.24 (−22.5, 21.7)	Exenatide			
2.04 (−18.6, 22.6)	2.22 (−19.8, 23.0)	Dulaglutide		
−9.31 (−32.7, 12.6)	−9.08 (−31.6, 11.3)	−11.4 (−32.9, 10.3)	Semaglutide	
2.85 (−12.2, 17.9)	3.11 (−13.1, 18.4)	0.749 (−13.8, 16.1)	12.3 (−2.86, 27.9)	Placebo

The league tables show the relative effects of each medication (the treatment on the column to the treatment of the row). The relative effects are measured as a difference in mean difference (DMD) with 95%CI for mean change in actual body weight (kg), waist circumference (cm), BMI, and BMI z score. Bold indicates statistical significance.

Supplemental Figure S5. League tables of safety analysis.

Dulaglutide	0.48 (0.03, 5.60)	0.71 (0.04, 12.93)	0.61 (0.03, 12.80)	2.09 (0.24, 22.76)
1.24 (0.03, 45.52)	Liraglutide	1.48 (0.27, 13.99)	1.28 (0.17, 18.37)	**4.31 (1.74, 19.72)**

1.13 (0.02, 68.34)	0.94 (0.05, 17.19)	Exenatide	0.86 (0.07, 11.72)	2.98 (0.64, 14.36)
0.89 (0.01, 62.53)	0.73 (0.02, 19.53)	0.79 (0.01, 36.59)	Semaglutide	3.38 (0.41, 23.68)
0.18 (0.01, 3.93)	**0.15 (0.02, 0.70)**	0.16 (0.01, 1.65)	0.20 (0.01, 3.97)	Placebo

Dulaglutide	0.67 (0.06, 4.70)	1.45 (0.13, 20.90)	1.29 (0.08, 17.03)	1.48 (0.22, 10.30)
2.66 (0.17, 63.21)	Liraglutide	2.18 (0.40, 25.63)	1.87 (0.29, 21.11)	2.22 (0.90, 9.43)
0.94 (0.03, 19.44)	0.37 (0.02, 3.07)	Exenatide	0.92 (0.05, 8.40)	1.04 (0.15, 4.69)
3.70 (0.09, 104.5)	1.40 (0.06, 14.74)	3.79 (0.18, 85.12)	Semaglutide	1.17 (0.19, 8.01)
1.20 (0.08, 14.62)	0.46 (0.07, 1.46)	1.23 (0.20, 9.52)	0.33 (0.03, 3.53)	Placebo

Dulaglutide	1.57 (0.22, 13.33)	1.62 (0.21, 10.44)	1.63 (0.18, 15.59)	1.64 (0.33, 9.12)
1.60 (0.05, 62.24)	Exenatide	1.04 (0.15, 4.65)	1.02 (0.14, 6.82)	1.05 (0.28, 3.49)
**3.665e^7 (8.033, 1.172e^{28})**	**2.382e^7 (5.214, 9.139e^{27})**	Liraglutide	1.01 (0.19, 6.68)	1.02 (0.42, 3.11)
0.76 (0.02, 27.45)	0.45 (0.01, 16.82)	**0.00 (0.00, 0.12)**	Semaglutide	1.02 (0.23, 4.58)
1.12 (0.09, 14.84)	0.68 (0.05, 8.94)	**0.00 (0.00, 0.12)**	1.47 (0.10, 24.69)	Placebo

Dulaglutide			
3.88 (0.54, 34.38)	Liraglutide		
4039. (1.01e^{-14}, 7.40e^{26})	898.4 (3.664$e^{-15,}$ 1.65e^{26})	Exenatide	
1.58 (0.23, 8.93)	**0.40 (0.14, 0.89)**	0.00 (2.42e^{-27}, 1.22e^{14})	Placebo

Nausea
Vomiting
Diarrhea
Abdominal pain
Headache
Injection reaction
Hypoglycemia

The league tables show the relative effects of each medication (the treatment on the column to the treatment of the row). The relative effects are measured as odds ratios for participants with nausea, vomiting, diarrhea, abdominal pain, headache, injection reaction, hypoglycemia, along with 95% CIs. Bold indicates statistical significance.
